# HER2 Score-Aware Virtual Immunohistochemistry via Non-Contrastive Multi-Task Translation

**DOI:** 10.3390/diagnostics16091319

**Published:** 2026-04-28

**Authors:** Hyunsu Jeong, Chiho Yoon, Jaewoo Kim, Eunwoo Park, Hyunhee Kim, Somang Park, Hyeon Gyu Kim, Chan Kwon Jung

**Affiliations:** 1 Graduate School of Artificial Intelligence, Pohang University of Science and Technology (POSTECH), Pohang 37673, Republic of Korea; 89douner@postech.ac.kr; 2 Department of Electrical Engineering, Pohang University of Science and Technology (POSTECH), Pohang 37673, Republic of Korea; ych000@postech.ac.kr; 3 Department of Convergence IT Engineering, Pohang University of Science and Technology (POSTECH), Pohang 37673, Republic of Korea; kimjaewoo@postech.ac.kr (J.K.); eunwoopark@postech.ac.kr (E.P.); hyunhee.kim@postech.ac.kr (H.K.); sompark05@postech.ac.kr (S.P.); 4 Department of VR Convergence Engineering, Duksung Women’s University, Seoul 01369, Republic of Korea; 5 Department of Hospital Pathology, Seoul St. Mary’s Hospital, and Cancer Research Institute, College of Medicine, The Catholic University of Korea, Seoul 06591, Republic of Korea

**Keywords:** image-to-image translation, virtual staining, SimSiam, non-contrastive learning, multi-task learning, HER2 score, immunohistochemistry

## Abstract

**Background/Objectives:** While human epidermal growth factor receptor 2 (HER2) immunohistochemistry (IHC) is pivotal for breast cancer management, its reliance on additional tissue processing beyond routine H&E staining remains a clinical burden. Although virtual staining offers a potential solution, current methods often fail to explicitly account for HER2 score-specific expression patterns. To address this gap, we developed a score-aware framework designed for the precise generation of virtual HER2 IHC images. **Methods:** We introduce the non-contrastive multi-task (NCMT) framework, which integrates negative-free patch alignment, style–content constraints, and auxiliary HER2 score supervision for high-fidelity H&E-to-IHC translation. For rigorous evaluation, the model was validated on the BCI dataset, utilizing an official split of 3896 training and 977 independent test images derived from 51 whole-slide images. **Results:** NCMT demonstrated superior virtual staining performance, achieving a Fréchet Inception Distance (FID) of 38.8, a Kernel Inception Distance (KID) of 5.6, and an average Perceptual Hash Value (PHV) of 0.439. In downstream HER2 scoring tasks, while virtual IHC images alone yielded an accuracy of 83.01%, the fusion of H&E and virtual IHC further elevated performance to 97.85% accuracy and a 98.23% F1 score. These findings suggest that our framework effectively preserves diagnostic features while providing complementary information to H&E-based morphological analysis. **Conclusions:** NCMT enables HER2 score-aware virtual IHC generation from H&E and can serve as a complementary tool for HER2 assessment in digital pathology.

## 1. Introduction

**Figure 1:**
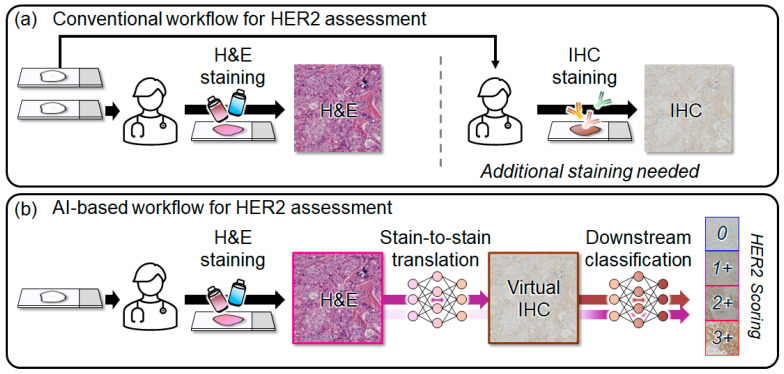
Conventional and AI-based workflows for HER2 immunohistochemistry (IHC) assessment in digital pathology. (**a**) Conventional workflow for HER2 assessment. (**b**) AI-based workflow for HER2 assessment.

Breast cancer is a leading cause of cancer-related mortality worldwide, underscoring the need for accurate pathological assessment [1,2,3,4,5,6,7]. In clinical practice, the diagnosis is initially established on biopsy tissue using hematoxylin and eosin (H&E) staining, which provides a morphological overview of tissue architecture and cellular atypia. However, treatment planning often requires molecular phenotype information that cannot be inferred from morphology alone. Immunohistochemistry (IHC) addresses this need by visualizing protein expression patterns in tissue [8]. Among the key IHC markers, hormone receptor (estrogen receptor and progesterone receptor) status and human epidermal growth factor receptor 2 (HER2) status are recognized as indispensable predictive and prognostic factors for therapeutic decision making in invasive breast carcinoma [9]. A positive HER2 test predicts benefit from HER2-targeted therapy, while HER2 overexpression reflects aggressive biology and poor prognosis without appropriate treatment. HER2 IHC scoring stratifies staining intensity and membranous patterns into ordinal scores (0, 1+, 2+, 3+), providing standardized criteria to guide clinical decision making in breast cancer treatment [10]. Nevertheless, performing IHC staining after H&E review requires additional tissue sections and laboratory processing, increasing cost and turnaround time within clinical workflows (Figure 1a) [11]. More broadly, modern clinical workflows are increasingly moving toward streamlined diagnostic strategies that minimize redundant procedures and resource utilization [12,13,14,15,16,17].

Recently, deep learning has been widely adopted in medical imaging to address the practical limitations of conventional diagnostic workflows [18,19,20,21,22,23,24,25,26,27,28,29,30,31,32,33]. In digital pathology, this trend extends to virtual stain-to-stain translation, where computational models generate a target staining domain (e.g., IHC) from an existing stained slide (e.g., H&E) [34,35,36,37]. Rather than performing additional chemical staining steps, stain-to-stain translation synthesizes the target stain from the available source image. By converting an H&E-stained slide into a virtual IHC-stained representation, this approach provides complementary biomarker information without requiring new tissue sections or extra laboratory processing. These advantages motivate virtual HER2 IHC generation as a scalable and cost-effective alternative for biomarker assessment, with the potential to facilitate IHC score-informed therapeutic decision making in breast cancer. At the same time, recent medical AI studies increasingly emphasize that model evaluation should extend beyond visual or predictive performance alone to also consider preservation of clinically relevant information for downstream decision support and classification [38,39,40,41,42].

Recent stain-to-stain translation methods employ patch-level contrastive objectives to explicitly maximize feature similarity between input and generated images [43,44,45]. In representative models such as CUT [46], a patch from the input image serves as an anchor. The objective maximizes similarity between the anchor and its corresponding positive patch, while minimizing similarity to negative patches sampled from other spatial locations. Typically, the positive is defined as the patch from the generated image at the same spatial location, while negatives are sampled from different spatial locations. Subsequent approaches such as ASP [47] and MDCL [48] refine this strategy by reweighting patch contributions or strengthening cross-domain feature alignment. However, this reliance on negative sampling introduces a fundamental limitation in histopathology. Pathological tissue exhibits repetitive and spatially correlated micro-structural patterns. Consequently, a patch sampled as a negative from a different location may still share highly similar morphological characteristics with the anchor and its positive counterpart. These false negatives generate conflicting learning signals because the objective pushes apart patches that may represent similar tissue content.

Non-contrastive objectives such as SimSiam provide an alternative that avoids explicit negatives [49]. SimSiam uses an asymmetric predictor with a stop-gradient target branch to learn from positive pairs only, which prevents representational collapse relying on negative sampling. Building on this principle, we propose non-contrastive multi-task translation (NCMT), a score-aware stain-to-stain framework for virtual IHC (Figure 1). Specifically, NCMT replaces contrastive learning with a patch-wise SimSiam loss tailored to stain-to-stain translation, introduces an asymmetric style–content (SC) loss to impose comparison-specific constraints, and incorporates an auxiliary HER2 score prediction task to encourage score-separable outputs. In downstream evaluation, we perform HER2 score classification using H&E and virtual IHC to assess its clinical utility. The primary contributions of this work are summarized below:

- We propose a patch-wise non-contrastive alignment strategy using SimSiam loss. This loss ensures structural consistency by focusing on positive pair alignment, thereby avoiding the false negative issues that arise when distinct patches share similar morphological features.
- An asymmetric style-content loss is introduced to establish an optimal balance between preserving morphological integrity of H&E and achieving realistic staining textures of IHC, enhancing the overall validity of the virtual immunohistochemistry.
- A score-aware multi-task learning framework is employed to jointly perform image translation and HER2 grade classification, enabling the model to explicitly capture grade-specific staining patterns.

## 2. Materials and Methods

### 2.1. Dataset

We train and evaluate NCMT on the BCI (Breast Cancer Immunohistochemical) dataset [50], which is well suited for score-aware virtual IHC because it provides registered H&E–IHC patch pairs together with a wide spectrum of HER2 expression level. This publicly available breast cancer histopathology dataset was introduced by researchers from Beijing University of Posts and Telecommunications and Capital Medical University, Beijing, China. According to the original dataset description, the whole-slide images were scanned using a Hamamatsu NanoZoomer S60 pathology section scanner at a scanning resolution of 0.46 μm/pixel. The BCI dataset has been widely adopted as a standardized benchmark in recent studies for virtual staining and HER2-related diagnostic tasks [51,52,53,54,55]. To facilitate a fair performance comparison, we strictly adhered to the official train/test dataset partition provided by the benchmark [50,55]. The dataset comprises 4873 H&E–IHC patch pairs extracted from 51 whole slide images. Each patch (1024 × 1024) is annotated with one of four HER2 IHC scores: 0 (*n* = 240), 1+ (*n* = 1154), 2+ (*n* = 2143), or 3+ (*n* = 1336), where higher scores indicate stronger and more extensive HER2 staining and typically correspond to higher expression. We use 3896 images for training and 977 images for testing.

### 2.2. Patch-Wise SimSiam Loss for Negative-Free Alignment

Conventional patch-wise contrastive learning encourages structural consistency by maximizing agreement between corresponding input and output patch representations. Its performance can be sensitive to how patch correspondences are defined and how sampling is performed when explicit negative patches are required. Prior work addressed these limitations by refining how negatives are selected and weighted, such as similarity-adaptive weighting based on patch similarity [47] or intra-domain hard negative mining [48]. In contrast, NCMT adopts SimSiam-style non-contrastive alignment [49] to avoid explicit negative sampling altogether. We align representations of positive patch pairs (e.g., corresponding patches between input–prediction or prediction–ground truth) using a prediction-based matching scheme with a stop-gradient target branch and an asymmetric predictor in the prediction branch. This asymmetric design stabilizes optimization and helps avoid degenerate constant representations without negatives. This is particularly appealing in histology, where spatial misalignment and repeated tissue patterns can cause near-corresponding patches to be treated as negatives, leading to unintended push-apart updates.

**Figure 2:**
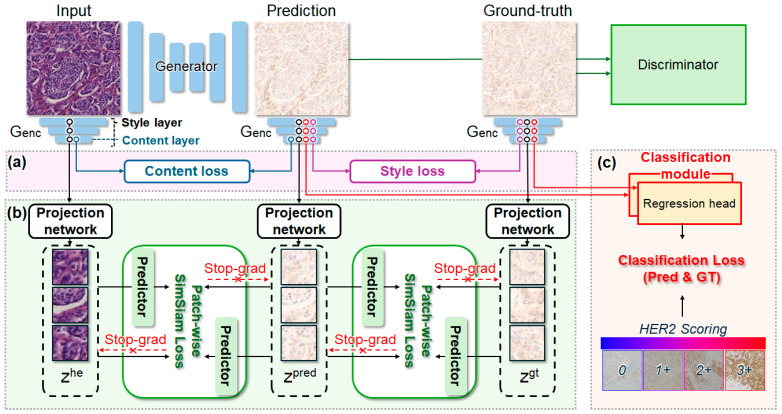
Proposed NCMT model architecture. (**a**) Style–content (SC) loss. (**b**) SimSiam loss. (**c**) Classification loss.

To enforce representation alignment without negative pairs, we introduce the patch-wise SimSiam loss (Figure 2b). We first apply it between the generated virtual IHC and the real IHC to make the generated output align with the ground truth. Let $x^{he}$, $x^{gen}$, and $x^{gt}$ denote the input H&E, generated IHC, and ground-truth IHC images, respectively. The generator encoder $G_{enc}(\cdot )$ is used as a feature extractor, yielding feature maps $\phi_{l}(\cdot )$ from selected layers l∈S. Following the PatchNCE pipeline, we randomly sample M=256 patch tokens and map each sampled feature to a latent embedding using a projection network f(⋅). For the sampled patches, we denote

(1) $$z^{t}=f\left(\phi_{l}\left(x^{t}\right)\right),\;t\in \left\{he,gen,gt\right\},$$

Following SimSiam, a predictor head h(⋅) produces $q_{i}=h(z_{i})$, and we prevent collapse by applying stop-gradient sg(⋅) to the target branch. The symmetrized patch-wise loss between the generated and ground-truth IHC is defined as

(2) $$L_{i}^{gt}=\frac{1}{2}D\left(q_{i}^{gen},sg(z_{i}^{gt})\right)+\frac{1}{2}D\left(q_{i}^{gt},sg(z_{i}^{gen})\right),\;D(u,v)=-\frac{u}{∥u{∥}_{2}}\cdot \frac{v}{∥v{∥}_{2}}.$$

We aggregate over patches using adaptive weights $w_{i}$ [47] to emphasize more reliable correspondences:

(3) $$L_{psm}^{gt}=\frac{1}{M}\sum_{i=1}^{M}w_{i}\;L_{i}^{gt},$$

Similarly, we apply the same formulation between $x^{gen}$ and $x^{he}$ to encourage structural consistency:

(4) $$L_{psm}^{he}=\frac{1}{M}\sum_{i=1}^{M}L_{i}^{he}.$$

The final patch-wise SimSiam loss is

(5) $$L_{psm}=L_{psm}^{gt}+L_{psm}^{he}.$$

### 2.3. SC Loss

While patch-level representation alignment helps preserve structural consistency, stain-to-stain translation also requires a careful balance between preserving content and expressing stain-specific style. To this end, we introduce an asymmetric SC loss that decouples content- and style-focused constraints according to the comparison objective (Figure 2a). Specifically, we enforce content consistency on the input–prediction pair to preserve tissue morphology on the H&E slide and style fidelity on the prediction–ground-truth pair to match the target IHC appearance.

Using the same encoder features $\phi_{l}(\cdot )$ extracted from $G_{enc}$ as in Equation (1), we define the content loss from a selected content layer c as

(6) $$L_{content}={∥\phi_{c}(x^{gen})-\phi_{c}(x^{he})∥}_{1},$$

and the style loss between $x_{gen}$ and $x_{gt}$ using Gram matrices G(⋅) over the style–layer set S as

(7) $$L_{style}=\frac{1}{|S|}\sum_{l\in S}{∥G\left(\phi_{l}(x^{gen})\right)-G\left(\phi_{l}(x^{gt})\right)∥}_{1}.$$

The final SC loss is

(8) $$L_{SC}=L_{content}+\beta \;L_{style},\;\beta =100.$$

We set β=100 to ensure that stain-specific color and texture cues are sufficiently enforced, since morphology-preserving constraints can otherwise dominate the optimization and reduce target-stain fidelity.

### 2.4. Multi-Task Learning

Even when the model is trained to follow the overall staining style, it can be guided to match dominant, common score patterns rather than faithfully encoding cues specific to each score. To explicitly encourage score-aware virtual IHC, we incorporate multi-task learning with an auxiliary score prediction objective (Figure 2c). In our setting, the HER2 score is treated as an ordinal severity signal and encoded as a continuous target value t∈{0, 0.33, 0.66, 1} corresponding to scores 0, 1+, 2+, 3+, respectively. We predict the HER2 score label (ordinal value) from intermediate encoder representations using the same multi-layer features $\left\{\phi_{l}(\cdot ){\}}_{l\in S}\right.$ as in the style term (Equation (7)). For each selected layer l∈S, we attach a lightweight regression head $g_{l}(\cdot )$ and compute a layer-wise HER2 score prediction

(9) $${\hat{t}}_{l}(x)=g_{l}\left(\phi_{l}(x)\right),$$

and the final score prediction is obtained by averaging across layers:

(10) $$\hat{t}(x)=\frac{1}{|S|}\sum_{l\in S}{\hat{t}}_{l}(x).$$

We supervise both the generated ($x^{gen}$) and ground-truth IHC ($x^{gt}$) using mean squared error (MSE):

(11) $$L_{cls}=MSE\left(\hat{t}(x^{gen}),\;t\right)+MSE\left(\hat{t}(x^{gt}),\;t\right).$$

This auxiliary objective both stabilizes the grading head on real IHC features and encourages NCMT to generate virtual IHC that retains score-dependent staining cues. The overall training objective is

(12) $$L_{total}=L_{gen}+\lambda_{psm}\;L_{psm}+\lambda_{SC}L_{SC}+\lambda_{cls}L_{cls}.$$

HER2 score labels are used only during training and are not provided at inference, so downstream classification reflects information encoded in the generated outputs rather than label leakage.

### 2.5. Downstream HER2 Score Classification

**Figure 3:**
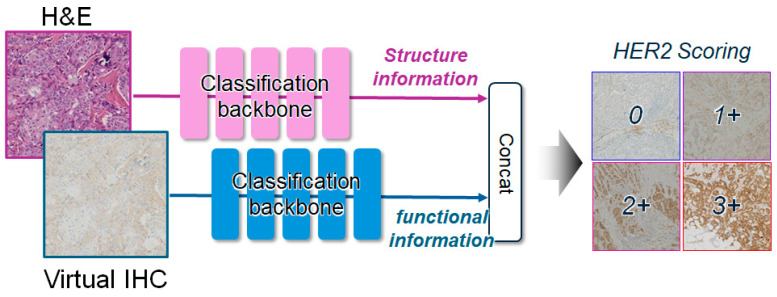
Dual-stream fusion classifier for HER2 immunohistochemistry (IHC) scoring.

To further assess the clinical potential of NCMT beyond visual translation quality, we conduct a downstream HER2 score classification (Figure 3). We train multiple standard classification backbones under two input settings, virtual IHC alone and a dual-stream fusion of H&E and virtual IHC, where H&E provides structural morphology and virtual IHC provides functional information. Functional information denotes molecular expression cues in IHC that are relevant to HER2 scoring. In the dual-stream setting, we extract feature embeddings from H&E and the generated virtual IHC using two encoders, concatenate the embeddings, and feed them to a fully connected classifier for HER2 score prediction:

(13) $$\hat{y}=FC\;\left(Concat(\left[f_{he}\left(x^{he}\right),f_{gen}\left(x^{gen}\right)\right]\right),$$

Here, $x^{he}$ and $x^{gen}$ denote the H&E and generated virtual IHC patches, $f_{he}$ and $f_{gen}$ are backbone-specific encoders, and Concat[⋅,⋅] denotes feature concatenation.

### 2.6. Implementation Details

The proposed NCMT was implemented using Python 3.9 and PyTorch 1.12. All experiments were conducted on a single NVIDIA RTX 3090 GPU. We utilized a Resnet-9 Blocks for the generator and a five-layer patch-generative adversarial network (GAN) [56] as the discriminator. Optimization was performed using the Adam optimizer with $\beta_{1}\;$= 0.5 and $\beta_{2}\;$= 0.999. During the training phase, input images were cropped to a resolution of 512 × 512. The models were trained for a total of 100 epochs with a batch size of 1. The initial learning rate was set to ${2\times 10}^{-4}$, with a linear decay schedule applied after the first 70 epochs. All compared models were trained using the same setting for a fair comparison; for MDCL, we instead adopted the original training configuration reported in its paper [48], as its performance is particularly sensitive to the NCE/ASP weights, and forcing our exact setting could yield an uninformative baseline.

For patch-level objectives, we extracted multi-layer encoder features using S={0, 4, 8, 12, 16} for SimSiam loss, style loss, and classification, and we set the content layer to c=12. For the Patch-wise SimSiam loss, we set the number of sampled patches M to 256 and used the lambda-linear scheme for the adaptive weights $w_{i}$ in Equation (3). In Equation (12), we set $\lambda_{psm}=10$, $\lambda_{SC}=1$, and $\lambda_{cls}=10$.

For the classification network, we trained the classifier for 100 epochs with input resolution 1024×1024, batch size 8, and learning rate $1\times 10^{-4}$. We used a five-fold data split during training to train five-fold-specific classification models. During training, classification was conducted using H&E and the generated IHC from the training set. For inference, we aggregated predictions from the five-fold-specific models using hard voting.

### 2.7. Evaluation Metrics

To quantitatively evaluate NCMT-generated virtual IHC, we report the Fréchet Inception Distance (FID) [57] and Kernel Inception Distance (KID) [58], which compare the feature–distribution gap between generated and real IHC images in the inception. In addition, we use the Perceptual Hash Value (PHV) to measure perceptual/content similarity between the generated image and its corresponding ground-truth IHC at four hierarchical feature levels (layers 1–4) [59]. We set the PHV threshold to T = 0.01, where lower values indicate better agreement. For downstream HER2 IHC score classification, we report standard multi-class performance metrics, including accuracy (ACC) and F1 score. To quantify statistical uncertainty, 95% confidence intervals were estimated using bootstrap resampling with 1000 iterations.

## 3. Results

### 3.1. Stain-to-Stain SOTA Model Comparison

**Figure 4:**
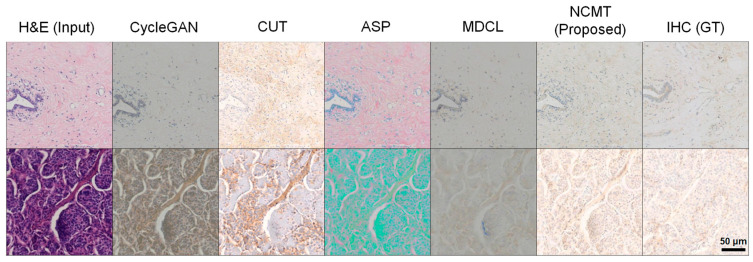
Qualitative comparison with SOTA stain-to-stain translation baselines.

To validate the effectiveness of NCMT for HER2 score-aware virtual IHC generation, we performed comparative experiments against state-of-the-art (SOTA) stain-to-stain baselines, including CycleGAN [60], CUT [46], ASP [47], and MDCL [48]. Figure 4 presents qualitative results across HER2 IHC score, and the corresponding quantitative results are summarized in Table 1.

In the qualitative comparisons (Figure 4), several baselines exhibit characteristic failure modes that directly affect score interpretation. In particular, contrastive learning-based models (CUT, ASP, and MDCL) often misestimate the target stain tone and luminance, producing outputs with miscalibrated intensity or biased color tones. In contrast, NCMT more reliably reproduces score-dependent staining intensity and spatial distribution patterns while preserving tissue morphology. This results in virtual IHC outputs that are visually closer to real IHC and more consistent for score assessment, with NCMT achieving the best overall performance across all metrics (FID = 38.8, KID = 5.6, and average PHV = 0.439).

### 3.2. Module Ablation Study

**Figure 5:**
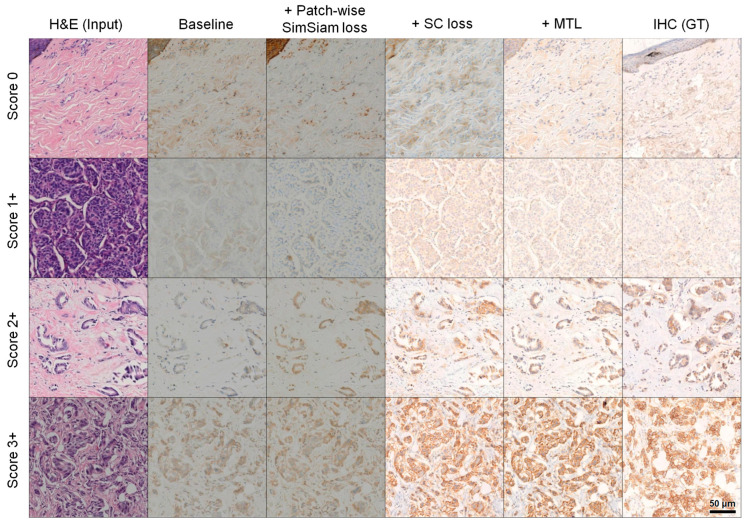
Qualitative results of the stain-to-stain module ablation study.

An ablation study was conducted to evaluate the contribution of each component in NCMT, as summarized in Figure 5 and Table 2. The analysis followed a progressive configuration: starting from the baseline framework, we sequentially added (1) the patch-wise SimSiam loss, (2) the asymmetric SC loss, and (3) the HER2 scoring multi-task learning (MTL) objective, and we evaluated performance on the total test set as well as score-wise subsets.

First, adding the patch-wise SimSiam loss generally improves performance across scores compared to contrastive baselines. In both quantitative metrics and qualitative comparisons, this results in more consistent preservation of local cellular and membranous staining patterns: blue-colored nuclear details are better maintained in no membranous staining (score 0) or faint incomplete membranous expression (score 1+), and the weak/moderate complete membranous signal in score 2+ is more faithfully reproduced without wash-out or over-saturation.

Adding the asymmetric SC loss yields a consistent improvement across HER2 IHC scores, with a marked gain on the total set: FID decreases by 6.4 and KID decreases by 5.9 compared to the SimSiam-only setting. As reflected in the qualitative results, combination of SimSiam with SC loss notably improves stain tone and brightness matching while maintaining structural integrity via its asymmetric design. However, it still has limitations in matching subtle staining variations in the ground truth.

Finally, incorporating the MTL objective achieves the best overall performance and the most real IHC–like outputs. With MTL, NCMT better captures score-specific staining characteristics, including membranous staining intensity and spatial distribution patterns that define HER2 scoring. This leads to consistent gains and the best results for every score in Table 2. Overall, compared to the configuration without MTL, the full model further improves the total performance, reducing FID by 2.3, KID by 1.4, and average PHV by 0.27, confirming that MTL provides additional supervision that strengthens HER2 score-aware separability and translation fidelity.

In addition, to assess the effect of spatial mismatch between serial tissue sections, we performed a controlled misalignment analysis by shifting the input H&E crop while keeping the target HER2 IHC crop fixed during training. As shown in Table 3, increasing offset magnitude progressively worsened FID, KID, and PHV avg., indicating that spatial mismatch acts as label noise and reduces marker-specific translation fidelity.

### 3.3. Classification

To further evaluate the clinical utility of HER2 score-aware virtual IHC, we performed a downstream HER2 score prediction study using five representative classification backbones: MaxViT-Tiny [61], EfficientNet-B0 [62], DenseNet-121 [63], Inception-v3 [64], and DeiT III-Small/16 [65]. For each backbone, we compared two input settings: virtual IHC alone and a fusion setting that jointly uses H&E and virtual IHC by concatenating their feature embeddings.

Using virtual IHC alone, the classifiers achieve moderate performance (ACC 56.91–83.01%), indicating that NCMT-generated virtual IHC retains score-relevant cues for HER2 scoring (Table 4). When H&E is fused with virtual IHC, we observe consistent gains in both ACC and F1 score for all backbones, with convolutional neural network (CNN) models reaching over 90% ACC. In contrast, vision Transformer (ViT)-style models improve but remain below 80% ACC even with fusion. Overall, the best performance is achieved by EfficientNet in the fusion setting (97.85% ACC and 98.23% F1), supporting the effectiveness of combining H&E morphology with NCMT-generated virtual IHC for score-aware HER2 assessment.

## 4. Discussion

HER2 IHC is routinely used to evaluate membranous HER2 protein expression, serving as a critical predictive biomarker for breast cancer treatment. However, performing IHC staining requires additional tissue sectioning and laboratory processing, resulting in increased turnaround time and cost due to the need for specialized reagents and instruments, technical expertise, and manual workflow steps. Recent advancements in contrastive learning have led to the development of style transfer frameworks capable of generating synthetic IHC images directly from H&E-stained slides. However, in histopathology, the morphological similarity between negative patches sampled from different spatial locations can lead to poorly differentiated features, potentially hindering the optimization and degrading training performance.

To overcome this limitation, we adopt a non-contrastive learning strategy that eliminates the need for negative patches. A qualitative review by pathologists confirmed that the generated virtual IHC preserves clinically meaningful membranous staining patterns consistent with HER2 expression. In SOTA model comparisons, NCMT achieves better qualitative and quantitative results than contrastive learning-based models that utilize negative patches for training. While CUT, ASP, and MDCL push anchors away from the negatives sampled at other locations, negative selection can be unreliable in pathology due to repeated structures and visually similar tissue patterns. By contrast, the proposed NCMT builds on a SimSiam-style non-contrastive alignment that learns from positive pairs only via an asymmetric predictor–target design with a stop-gradient, preventing representation collapse without negative sampling and reducing adverse updates from false negatives. Consistent with this stable alignment, NCMT more effectively preserves local cellular and membranous staining patterns with reduced patch-wise inconsistency, and its global stain calibration (tone and luminance) is further strengthened by the asymmetric SC loss, which explicitly promotes appearance matching while preserving morphology. Finally, the HER2 scoring MTL objective provides direct score supervision, helping to improve score-aware staining representation and yielding the best overall performance in our ablation study.

To assess clinical utility, we test whether NCMT-generated virtual HER2 IHC can support HER2 score recognition. While virtual IHC alone preserves score-related expression cues, HER2 score prediction performance remains limited. When we fuse H&E morphology with virtual IHC features, performance generally improves across backbones. Because HER2 IHC scoring depends on the membranous staining pattern, H&E morphology provides useful context for interpretation, such as tumor cellularity, tissue architecture, and heterogeneity; therefore, combining H&E with virtual IHC leads to more reliable IHC scoring. This finding suggests that virtual IHC does not replace morphological assessment but rather complements it by providing additional information related to protein expression. Such a hybrid approach may be particularly beneficial in challenging cases, including borderline or equivocal (2+) interpretations, where subtle differences in membranous staining patterns are critical for accurate assessment. However, Transformer-based models (MaxViT/DeiT) show slower gains than CNNs even with fusion, consistent with the higher data demand of ViT-style architectures under limited training sets.

While our proposed model offers significant potential for digital pathology, several critical challenges remain. The reliance on serial sections introduces inherent morphological shifts and physical distortions, precluding precise pixel-to-pixel registration between H&E and IHC slides. This lack of exact alignment complicates the curation of high-quality ground truth, particularly as local discrepancies near tissue boundaries and glandular structures introduce label noise. Such noise may force the model to prioritize coarse contextual patterns over genuine marker-related morphology. Consequently, future research should focus on robust learning strategies—such as patch-level soft matching [66], uncertainty-aware loss functions [67], and deformation-tolerant modules [68]—to mitigate registration misalignment and enhance generalization across diverse staining protocols and imaging domains. In addition, integrating clinically curated datasets with external controls would further strengthen the reliability of the proposed approach.

From a clinical perspective, virtual IHC should be interpreted as a complementary tool rather than a replacement for conventional staining. Its potential applications may include pre-screening, decision support, and workflow optimization, particularly in settings with limited resources or high case volumes. In the broader context of digital pathology, such approaches may facilitate earlier access to biomarker-related information during initial slide review and reduce dependency on sequential staining procedures.

Recent clinical trends have increasingly emphasized the significance of the HER2-low and HER2-ultralow landscape. While traditional assessments focus on distinguishing positive (3+) from negative (0/1+) cases, the ability to discriminate between a complete absence of HER2 expression (0) and minimal membrane staining (0+) has become critical for treatment planning. However, in this study, we utilized the BCI dataset to enable objective performance comparison with existing SOTA frameworks, which follow a four-tier labeling scheme: 0, 1+, 2+, and 3+. Consequently, the current model does not explicitly distinguish the 0+ subcategory. Future research should therefore focus on developing more granular virtual staining models trained on datasets that capture these subtle expression patterns, thereby further enhancing clinical utility.

Furthermore, because HER2 status involves molecular-level protein expression that may fluctuate independently of histological features, H&E-based models face the risk of producing visually plausible but semantically inaccurate results. This challenge is magnified by intratumoral heterogeneity and pre-analytic variables—such as fixation time and antibody clones—which complicate the inference of absolute expression levels from morphology alone. Consequently, the clinical value of virtual staining rests on its quantitative concordance—measured through intensity correlation and scoring consistency—rather than mere stylistic similarity. This necessitates multimodal frameworks that integrate auxiliary evidence, such as transcriptomic data and molecular profiling information, to constrain generation toward biologically valid patterns [69]. Additionally, incorporating uncertainty estimation to flag low-confidence outputs is essential for ensuring the safe clinical application of virtual IHC.

In conclusion, this study demonstrates that incorporating clinically structured supervision into virtual staining frameworks enhances both representation quality and downstream utility. By bridging morphological and molecular information within a unified model, the proposed approach represents a meaningful step toward clinically integrated artificial intelligence systems in digital pathology.

## Figures and Tables

**Table 1:** Quantitative comparison with SOTA stain-to-stain translation baselines. The KID values in the table are scaled by a factor of 1000.

Model	FID ↓	KID ↓	PHV Layer1 ↓	PHV Layer2 ↓	PHV Layer3 ↓	PHV Layer4 ↓	PHV Avg. ↓
CycleGAN	87	51.3	0.552	0.464	0.33	0.804	0.538
ASP	221	103	0.659	0.609	0.409	0.856	0.633
CUT	56.3	17.3	0.637	0.506	0.277	0.757	0.544
MDCL	50.7	14.4	0.510	0.398	0.238	0.739	0.471
NCMT (Proposed)	**38.8**	**5.6**	**0.446**	**0.364**	**0.227**	**0.717**	**0.439**

↓ indicates that lower values are better. Bold values indicate the best score for each metric.

**Table 2:** Quantitative results of the stain-to-stain module ablation study. The final proposed model (NCMT) is highlighted in yellow. The KID values in the table are scaled by a factor of 1000.

HER2 Score	Patch-Wise SimSiam Loss	SC Loss	MTL	FID ↓	KID ↓	PHV Layer1 ↓	PHV Layer2 ↓	PHV Layer3 ↓	PHV Layer4 ↓	PHV Avg. ↓
0	X	X	X	171.8	12.6	0.675	0.48	0.26	0.751	0.541
	O	X	X	173.4	10.2	0.648	0.446	0.253	0.757	0.526
	O	O	X	171.8	7.0	0.598	0.442	0.249	0.763	0.513
	O	O	O	**146.9**	**1.6**	**0.588**	**0.402**	**0.237**	**0.723**	**0.488**
1+	X	X	X	84.9	17.0	0.52	0.373	0.22	0.723	0.459
	O	X	X	84.2	13.7	0.523	0.365	0.227	0.717	0.458
	O	O	X	75.9	10.4	0.479	0.384	0.22	0.718	0.45
	O	O	O	**67.7**	**3.9**	**0.407**	**0.315**	**0.197**	**0.687**	**0.402**
2+	X	X	X	60	11.7	0.493	0.379	0.225	0.723	0.455
	O	X	X	57.9	10.7	0.494	0.372	0.226	0.718	0.452
	O	O	X	58.3	9.3	0.493	0.406	0.235	0.723	0.464
	O	O	O	**50.8**	**6.5**	**0.449**	**0.37**	**0.223**	**0.707**	**0.437**
3+	X	X	X	129.9	42.5	0.507	0.44	0.275	0.782	0.501
	O	X	X	122.4	42.6	0.515	0.435	0.274	0.771	0.499
	O	O	X	115.1	26.4	**0.455**	0.409	0.27	0.771	0.476
	O	O	O	**109.6**	**24.1**	0.457	**0.394**	**0.261**	**0.763**	**0.469**
Total	X	X	X	50.7	14.4	0.51	0.398	0.238	0.739	0.471
	O	X	X	47.5	12.9	0.513	0.39	0.24	0.733	0.469
	O	O	X	41.1	7.0	0.484	0.403	0.24	0.736	0.466
	O	O	O	**38.8**	**5.6**	**0.446**	**0.364**	**0.227**	**0.717**	**0.439**

↓ indicates that lower values are better. Bold values indicate the best score for each metric.

**Table 3:** Quantitative analysis of NCMT under controlled crop misalignment during training. Results at each offset magnitude (0, 8, 16, and 24 pixels) were averaged over four directions (+*x*, −*x*, +*y*, −*y*).

Offset Magnitude (Pixels)	FID ↓	KID ↓	PHV Avg. ↓
0	**38.8**	**5.6**	**0.439**
8	42.05	7.2	0.455
16	42.77	7.7	0.445
24	46.68	9.3	0.464

↓ indicates that lower values are better. Bold values indicate the best score for each metric.

**Table 4:** HER2 score classification results across backbones for virtual IHC only and H&E–virtual IHC fusion. All values are presented as percentages, and 95% bootstrap confidence intervals are provided in parentheses.

Model	Virtual IHC		H&E + Virtual IHC	
	ACC ↑ (95% CI)	F1 ↑ (95% CI)	ACC ↑ (95% CI)	F1 ↑ (95% CI)
DeiT	56.91 (53.74–60.08)	42.45 (39.93–44.85)	69.19 (66.43–72.06)	61.59 (57.13–65.54)
MaxViT	69.50 (66.33–72.16)	56.77 (52.59–60.88)	77.28 (74.62–79.94)	71.38 (67.19–75.10)
Inception	65.92 (62.54–68.78)	61.16 (56.36–65.28)	90.38 (88.54–92.22)	89.62 (87.12–92.22)
DenseNet	74.82 (72.05–77.48)	68.66 (63.61–72.86)	94.27 (92.73–95.70)	93.57 (91.51–95.38)
EfficientNet	**83.01 (80.55–85.47)**	**80.62 (76.62–84.09)**	**97.85 (96.93–98.77)**	**98.23 (97.44–99.03)**

↑ indicates that higher values are better. Bold values indicate the best score for each metric.

## Data Availability

The data used in this study are publicly available. The BCI dataset can be accessed via the project homepage (https://bupt-ai-cz.github.io/BCI/) (accessed on 24 April 2026).

## Abbreviations

The following abbreviations are used in this manuscript:

ACC	Accuracy
BCI	Breast Cancer Immunohistochemical
CNN	Convolutional Neural Network
FC	Fully Connected
FID	Fréchet Inception Distance
GAN	Generative Adversarial Network
H&E	Hematoxylin and Eosin
HER2	Human Epidermal Growth Factor Receptor 2
IHC	Immunohistochemistry
KID	Kernel Inception Distance
MSE	Mean Squared Error
MTL	Multi-Task Learning
NCMT	Non-Contrastive Multi-Task Translation
PHV	Perceptual Hash Value
SC loss	Style–Content Loss
SOTA	State-of-the-Art
ViT	Vision Transformer
